# Systematic Review of All-Arthroscopic Versus Mini-Open Repair of Rotator Cuff Tears: A Meta-Analysis

**DOI:** 10.1038/srep22857

**Published:** 2016-03-07

**Authors:** Rongzhong Huang, Sanrong Wang, Yule Wang, Xiaoxia Qin, Yang Sun

**Affiliations:** 1 Department of Rehabilitation Medicine, the Second Affiliated Hospital of Chongqing Medical University, Chongqing, China; 2 Institute of Ultrasound Imaging, the Second Affiliated Hospital of Chongqing Medical University, Chongqing, China

## Abstract

The objective of this study was to compare outcomes in patients with rotator cuff tears undergoing all-arthroscopic versus mini-open rotator cuff repair. A systematic review and meta-analysis of outcomes of all-arthroscopic repair versus mini-open repair in patients with rotator cuff repair was conducted. Studies meeting the inclusion criteria were screened and included from systematic literature search for electronic databases including Medline, Embase, Cochrane CENTRAL, and CINAHL library was conducted from 1969 and 2015. A total of 18 comparative studies including 4 randomized clinical trials (RCTs) were included. Pooled results indicate that there was no difference in the functional outcomes, range of motion, visual analog scale (VAS) score, and short-form 36 (SF-36) subscales. However, Constant-Murley functional score was found to be significantly better in patients with mini-open repair. However, the results of the review should be interpreted with caution due to small size and small number of studies contributing to analysis in some of the outcomes. All-arthroscopic and mini-open repair surgical techniques for the management of rotator cuff repair are associated with similar outcomes and can be used interchangeably based on the patient and rotator tear characteristics.

Rotator cuff tears are common amongst the elderly and athletes. Surgery is usually required to help regain the muscle strength, function and flexibility of the shoulder, and to relieve the pain. There are many ways of repairing the rotator cuff tears, including arthroscopy, open surgery, or a combination of both. Clinical guidelines recommend using open surgery, mini-open surgery or arthroscopy for a full-thickness tear accessible to direct repair by suture^1^. Surgery has been found to be associated with better results compared to non-surgery^2^. Although, mini-open rotator cuff repair and arthroscopic repair are commonly performed for the treatment of rotator cuff tears, with comparable results; however, there is uncertainty on the long term outcomes using the two techniques^3^.

## Methods

### Study design

A systematic review was conducted in adult patients with rotator cuff tears other than massive or irreparable tears to compare clinical outcomes of patients undergoing all-arthroscopic versus mini-open rotator cuff repair. The review was conducted and reported according to the Preferred Reporting Items for Systematic Reviews and Meta-analysis (PRISMA) guidelines.

### Data sources

A systematic literature search of electronic databases for relevant studies between 1963 to May 2015 was conducted through Embase^®^, MEDLINE^®^, Cochrane CENTRAL, and CINAHL. Studies published in English language were identified using search terms like ‘rotator cuff’, ‘arthroscopy’, ‘mini-open’, and ‘supraspinatus’.

### Study eligibility

Studies were screened based on the predefined inclusion criteria. Comparative studies reporting all-arthroscopic rotator cuff repair (RCR) (no concurrent acromioplasty, superior labrum anterior to posterior [SLAP] or other procedures) versus mini-open RCR (no concurrent procedures) were included. Additionally, studies reporting sub-group data for patients of interest to the review were also included. Relevant outcomes of arthroscopic rotator cuff repair (ARCR) or mini-open rotator cuff repair (MRCR) included functional scores (University of California Los Angeles [UCLA] shoulder score, American Shoulder and Elbow Surgeons [ASES] shoulder outcome score, Constant-Murley scores), range of motion (abduction, forward flexion, external rotation); pain visual analog scale (VAS) score, and complications (retear, adhesive capsulitis). No restriction was employed to study design, with both retrospective and prospective cohort studies were included, except for case studies.

### Data collection

Bibliographic details and abstracts of all citations were retrieved through database searches. A team of independent reviewers specialized in evidence-based medicine determined the eligibility of each publication. Citations were initially screened on the basis of title/abstract supplied with each citation by applying the defined set of eligibility criteria described above. Duplicates of citations (due to overlap in the coverage of the databases) were excluded at this stage. Full text copies were ordered for studies that potentially met the eligibility criteria. The eligibility criteria were then applied to the full-text publications, with each publication being reviewed by an independent two review process.

Data was extracted from the full-text articles of included studies using a specifically designed data extraction grid. Only one dataset per study was compiled from all publications related to that study in order to avoid duplication of data. Outcome data from eligible studies were extracted from the latest time point in all trials.

### Study quality

Study quality was assessed using Newcastle-Ottawa Quality Assessment Scale for cohort studies and Cochrane Collaboration’s tool for assessing risk of bias for randomized controlled trials.

### Statistical analysis

Results will be expressed as mean differences for continuous outcomes (standardized vs. weighted to be determined by available data); and the appropriate ratio/difference for dichotomous outcomes as determined by available data. For pooled analyses and forest plot generation, we used Comprehensive Meta-Analysis software. To test the robustness of our results, we will perform sensitivity analyses to be determined by the available data. Random effects models will be used, as will appropriate tests for heterogeneity.

## Results

### Identification of relevant studies

**Figure 1:**
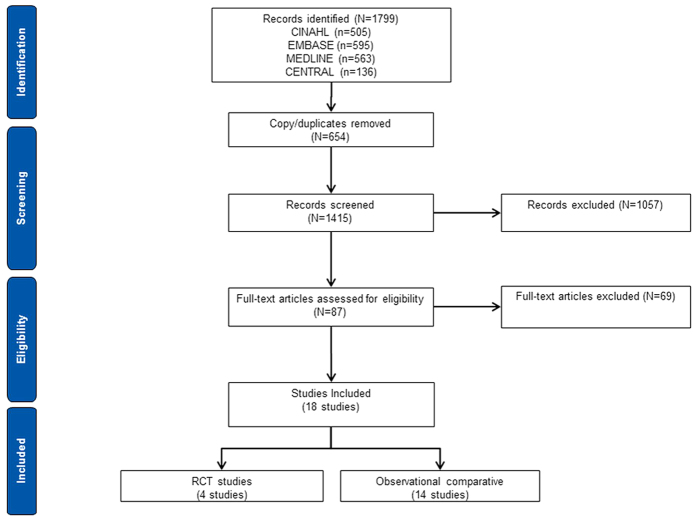
Trial flow of included studies.

Literature search yielded 1799 studies, of which 87 potentially relevant full-text articles were identified for detailed evaluation. Following detailed screening, 18 studies evaluating arthroscopy and mini-open repair for rotator-cuff repair were included in the review (Fig. 1). The included evidence was based on comparative studies assessing clinical outcomes or providing sub-group data on outcomes of interest in patients with rotator-cuff tear.

The list of studies included in the review along with study characteristics is presented in Table 1. Of the 18 included studies, 7 studies were conducted in the USA, three in South Korea, two in Germany, and one each in UK, Turkey, France, Italy, China, and the Netherlands. Of the included studies, four were RCT and 14 were observational studies over a data collection period from 2000 to 2014.

### Patient demographics

The summary of patient demographics for all included studies is presented in Table 1. Preoperative patient characteristics did not show any significant difference between these two groups with respect to the number of patients, gender and age.

### Outcome Measurements

The results of standardized mean difference (SMD) and 95% confidence interval (CI) for each comparison were shown in Table 2. Data at the study endpoint was pooled directly without stratifying for the study period due to considerable variability in the time period of follow-up in the included studies. Analyzable data was only reported in limited studies, which might contribute bias to our final results.

### Functional Results (UCLA Score, ASES Score, Constant Score, SST, DASH)

**Figure 2:**
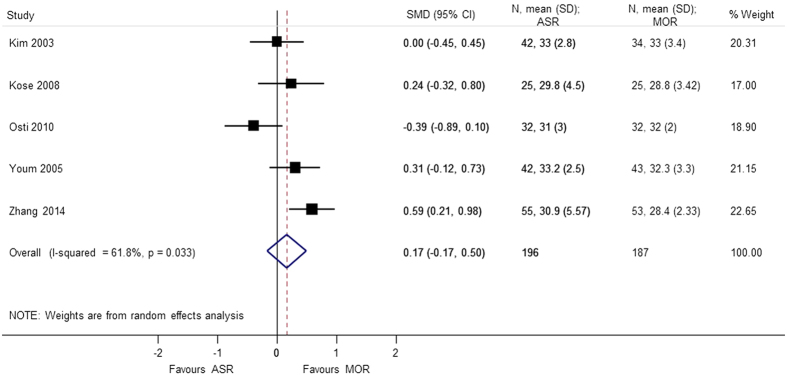
Forest plot showing the SMD (standardized mean difference) and 95% CI for UCLA (University of California Los Angeles) after surgery.

**Figure 3:**
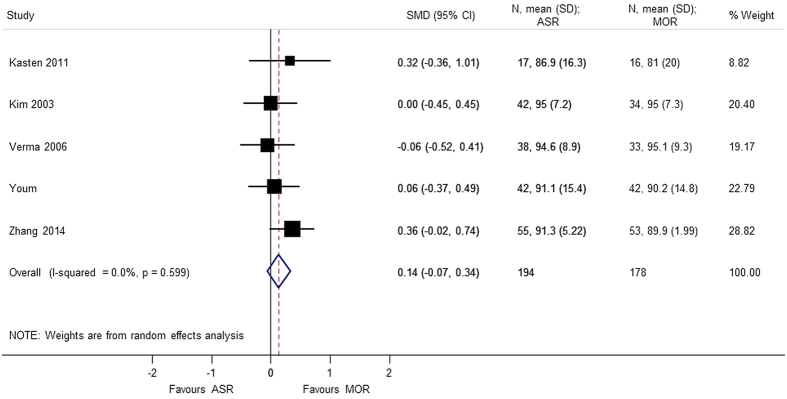
Forest plot showing the SMD (standardized mean difference) and 95% CI for ASES (American Shoulder and Elbow Surgeons) after surgery.

**Figure 4:**
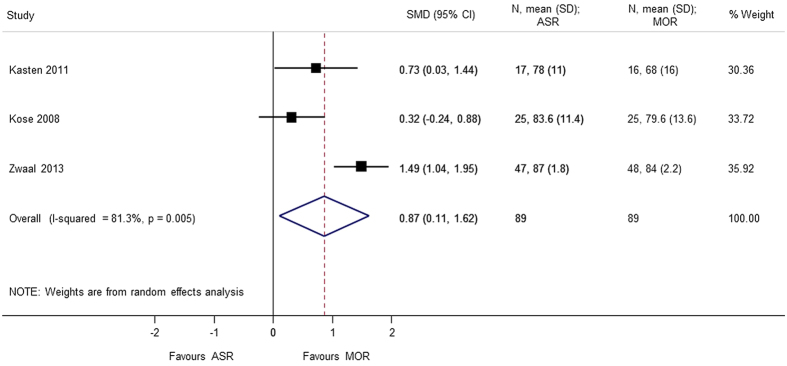
Forest plot showing the SMD (standardized mean difference) and 95% CI (Confidence Interval) for constant score after surgery.

**Figure 5:**
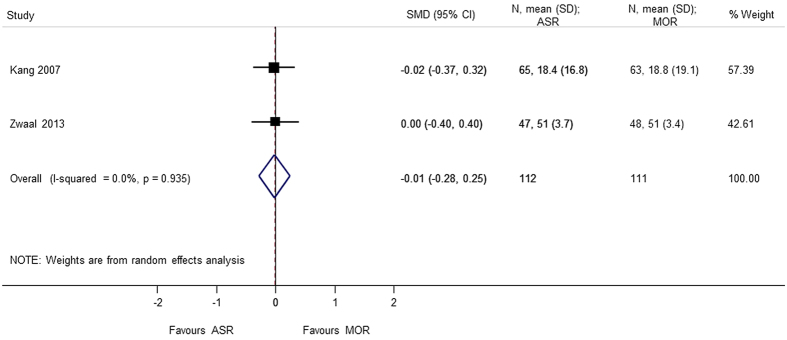
Forest plot showing the SMD (standardized mean difference) and 95% CI (Confidence Interval) for DASH (Disabilities of the Arm, Shoulder and Hand) score after surgery.

**Figure 6:**
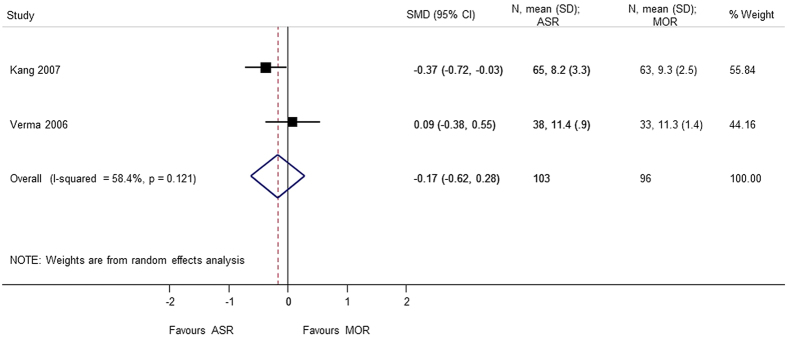
Forest plot showing the SMD (standardized mean difference) and 95% CI (Confidence Interval) for SST (Simple Shoulder Test) after surgery.

Nine studies^4^,^5^,^6^,^7^,^8^,^9^,^10^,^11^,^12^ using different score systems were involved when comparing the function score between two groups. Constant score was significantly better in mini-open repair group (SMD = 0.865 95% CI 0.109, 1.621); p = 0.025. However, there was considerable heterogeneity in the results of Constant score and sensitivity analysis was conducted after removing Zwaal *et al*.^9^, and the results were still found to be significant (SMD = 0.477 95% CI 0.039, 0.915); p = 0.033. For the remaining functional scores; there was no difference between the arthroscopic group and mini-open repair group, at different periods of follow-up; UCLA (SMD = 0.165 95% CI −0.166, 0.497), ASES (SMD = 0.136 95% CI −0.068, 0.340), disabilities of the arm, shoulder and hand (DASH) (SMD = −0.013 95% CI −0.275, 0.250), and simple shoulder test (SST) (SMD = −0.171 95% −0.620, 0.278) (Figs 2, 3, 4, 5, 6). Several studies, in which only mean value was reported, were not included in the analysis. However, authors in these studies gave similar results as our outcome.

### Range of Motion (Forward flexion, External Rotation, Internal rotation, Abduction)

**Figure 7:**
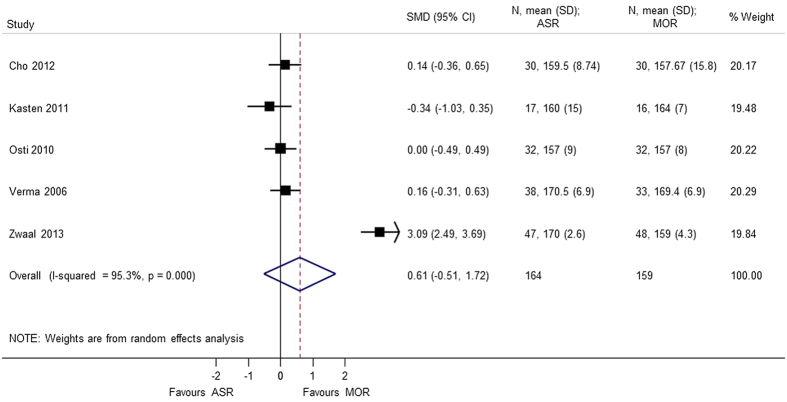
Forest plot showing the SMD (standardized mean difference) and 95% CI (Confidence Interval) for forward flextion after surgery.

**Figure 8:**
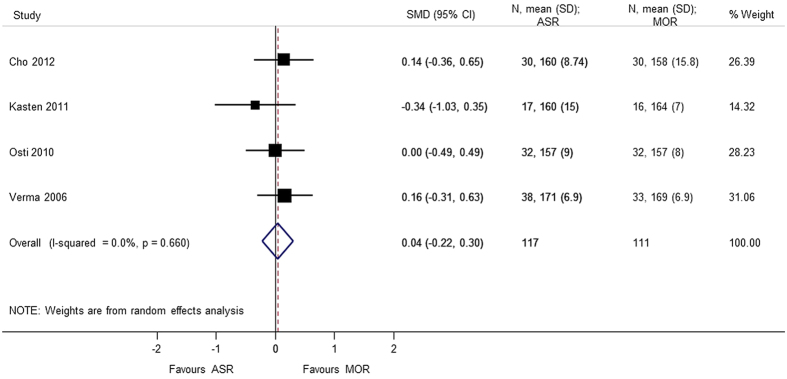
Forest plot showing the SMD (standardized mean difference) and 95% CI (Confidence Interval) for forward flextion after surgery (sensitivity analysis).

**Figure 9:**
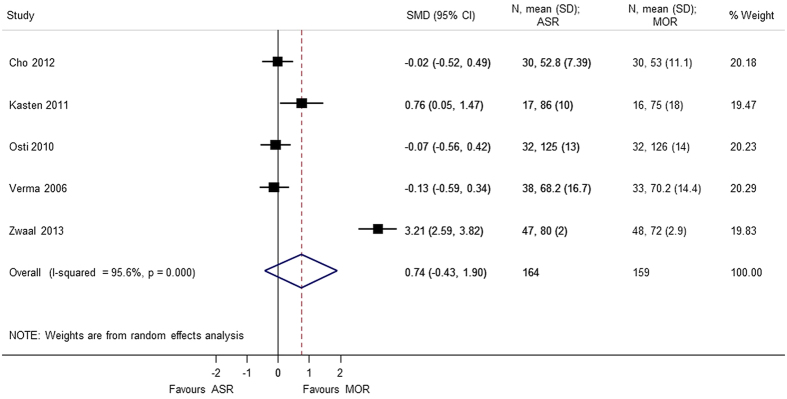
Forest plot showing the SMD (standardized mean difference) and 95% CI (Confidence Interval) for external rotation after surgery.

**Figure 10:**
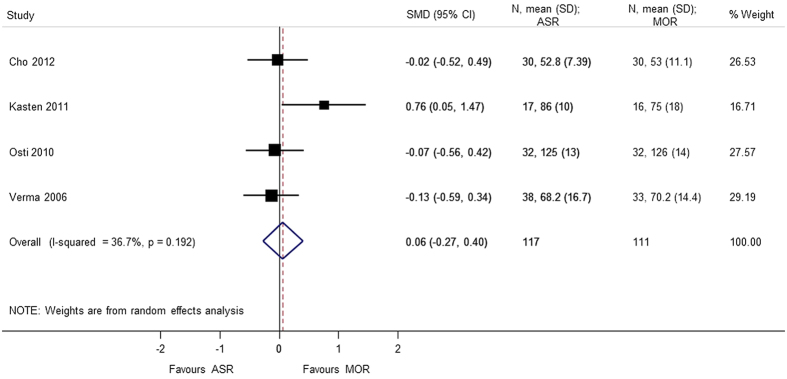
Forest plot showing the SMD (standardized mean difference) and 95% CI (Confidence Interval) for external rotation after surgery (sensitivity analysis).

**Figure 11:**
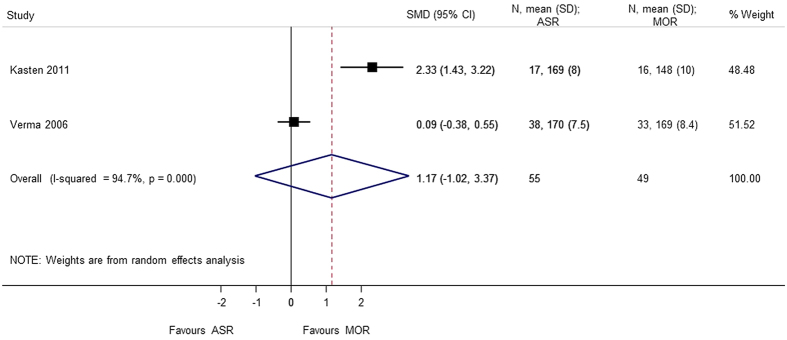
Forest plot showing the SMD (standardized mean difference) and 95% CI (Confidence Interval) for abduction after surgery.

**Figure 12:**
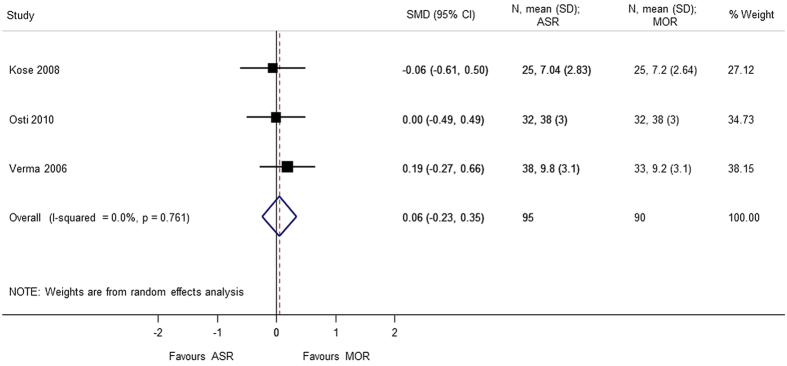
Forest plot showing the SMD (standardized mean difference) and 95% CI (Confidence Interval) for internal rotation after surgery.

A total of 6 studies^5^,^7^,^8^,^9^,^10^,^13^ provided analyzable data for postoperative range of motion (Forward flexion, External rotation, Internal rotation, Abduction) with 373 patients. No statistical difference was observed in forward flexion (SMD = 0.608 95% CI −0.506, 1.722), external rotation (SMD = 0.740 95% CI −0.426, 1.905), internal rotation (SMD = 0.058 95% CI −0.231, 0.347), and abduction (SMD = 1.174 95% CI −1.019, 3.367). During analysis, heterogeneity (p < 0.0001) of the combined data was found for abduction, external rotation, and forward flexion. A sensitivity analysis was performed by removing the outliers. The heterogeneity decreased on removing the confounding study, Zwaal *et al*.^9^ and the results were consistent with the primary analysis with no statistical significant difference between arthroscopic repair and mini-open repair (Figs 7, 8, 9, 10, 11, 12). The heterogeneity could be explained by the fact that patients with simultaneous lesions of the shoulder were excluded in Zwaal *et al*.^9^.

### Quality of life

Very few studies investigated the impact of arthroscopic and mini-open repair techniques on postoperative quality of life. SF-36, VAS, SF-12.

### VAS Score (Pain, Function)

**Figure 13:**
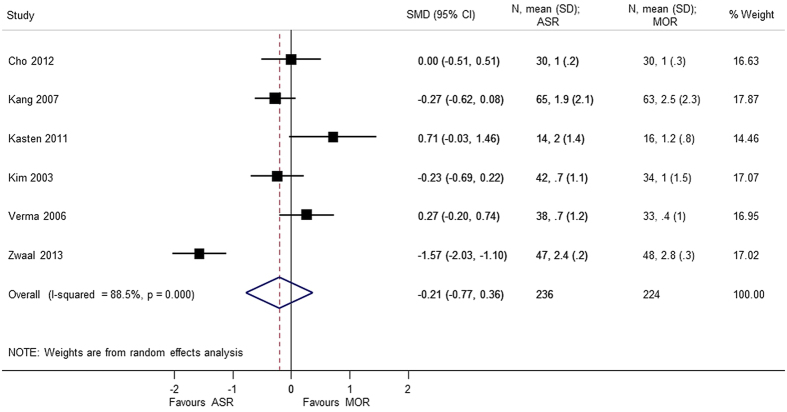
Forest plot showing the SMD (standardized mean difference) and 95% CI (Confidence Interval) for VAS (Visual Analog Scale) (pain) after surgery.

**Figure 14:**
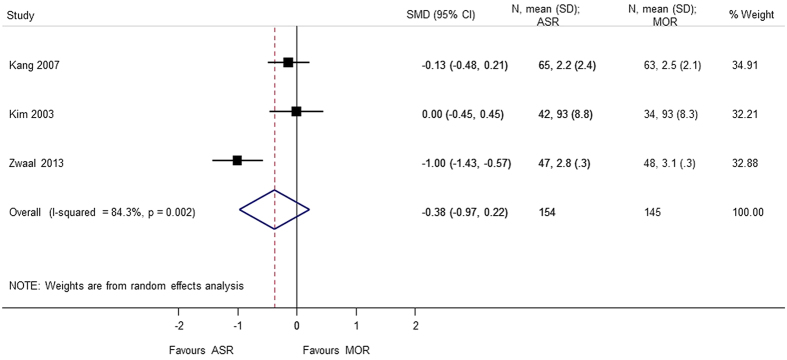
Forest plot showing the SMD (standardized mean difference) and 95% CI (Confidence Interval) for VAS (Visual Analog Scale) (function) after surgery.

**Figure 15:**
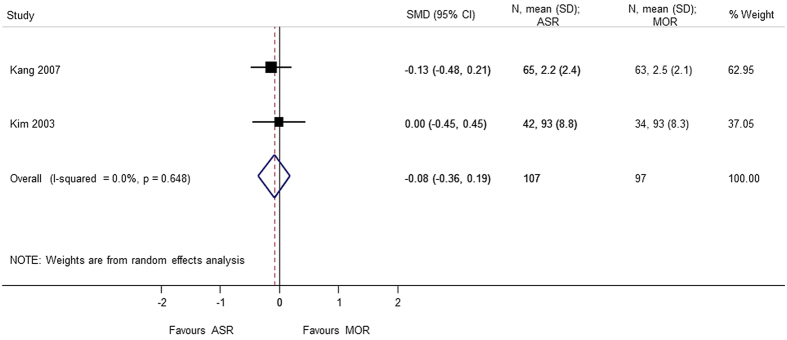
Forest plot showing the SMD (standardized mean difference) and 95% CI (Confidence Interval) for VAS (Visual Analog Scale) (function) after surgery (sensitivity analysis).

Six studies^4^,^5^,^6^,^9^,^10^,^13^ totaling to 460 patients were included for analysis of pain score and function using the visual analog scale (VAS). No significant difference were found between arthroscopic repair and mini-open repair on the VAS pain scale (SMD = −0.206 95% CI −0.775, 0.364) and VAS function scale (SMD = −0.084 95% CI −0.359, 0.192). A heterogeneity (p < 0.05) was found in the VAS analysis, and sensitivity analysis was performed by excluding the study by Zwaal *et al*.^9^. The reason might be attributed to arthroscopic development as discussed earlier (Figs 13, 14, 15).

### SF-36 (Bodily Pain, Role-Physical)

**Figure 16:**
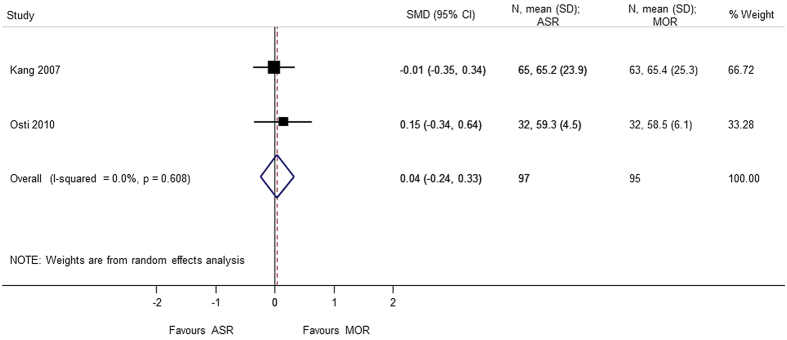
Forest plot showing the SMD (standardized mean difference) and 95% CI (Confidence Interval) for SF-36 (Short-Form 36) (bodily pain) after surgery.

**Figure 17:**
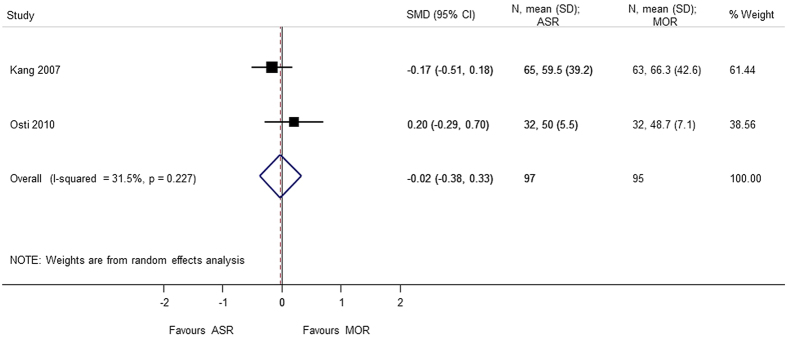
Forest plot showing the SMD (standardized mean difference) and 95% CI (Confidence Interval) for SF-36 (Short-Form 36) (role physical) after surgery.

In addition to the VAS score, analysis on the SF-36 subscales of role-physical and bodily pain was also performed, to check whether the results were consistent across the two scales for the similar outcomes. Two studies^4^,^8^ with 192 patients contributing to the analysis of SF-36 subscales were included. There was no statically significant difference in the arthroscopic repair and mini-open repair on the bodily-pain (SMD = 0.044 95% CI −0.239, 0.327) and role-physical (SMD = −0.023 95% CI −0.377, 0.331) subscales of SF-36. The results are consistent with the VAS pain and VAS function scales (Figs 16 and 17).

## Discussion

To our knowledge, this is most up-to-date systematic review including 18 studies, including both randomized and observational studies comparing arthroscopic repair with mini-open rotator cuff repair. Earlier conducted systematic reviews focus on specific study designs, RCTs^14^ or observational study design^15^. While some other reviews include limited publications, 5 studies^16^ and 12 studies^17^.

The results of our review are consistent with the previously conducted systematic reviews^14^,^15^,^16^,^17^, concluding that the two techniques (mini-open rotator cuff repair and arthroscopic repair) have similar outcomes and can be considered as alternative treatment options. However, the result of our meta-analysis show that the Constant-Murley score (CMS) was significantly better in the mini-open repair group compared to all arthroscopic repair. CMS is a 100-points scale composed of four subscales: pain (15 points), activities of daily living (20 points), strength (25 points) and range of motion: forward elevation, external rotation, abduction and internal rotation of the shoulder (40 points). On a 100-points scale, higher score is related to higher quality of the function^18^.

Tear size is an important factor for achieving satisfactory results, with more patients with large or massive cuff tears obtaining unsatisfactory response outcomes^19^. Zhang *et al*. noted that patients treated with arthroscopic group displayed better shoulder strength but a significantly higher retearing rate as compared to mini-open group at 24-month follow-up^12^. For full-thickness tears, retearing rates were 74% for the arthroscopic group and 35% for the mini-open group (p < 0.05). For partial-thickness tears, no significant difference was detected^12^. Kim *et al*. conclude that surgical outcomes depend upon the size of the tear, rather than the method of repair^6^. The operative time for arthroscopic repair was also significantly longer than that for mini-open repair^4^.

In a study by Verma *et al*., there was no difference in the outcome measure for VAS (pain) and ASES score between the intact and failed repair group, indicating that excellent symptomatic relief can be achieved regardless of tendon healing. However, significant differences existed between intact and failed repairs in the restoration of forward flexion, showing an adequate repair remains vital, if strength is to be restored^10^.

Surgical technique had an impact on return to work, with an open procedure (66% patients) being advantageous compared to arthroscopic repair (45.3%) and mini-open repair (41.6%) (p = 0.004). However, there was no significant difference in the time away from work between the groups, even if it was slightly longer for open procedures^20^.

Warner *et al*. tested two hypotheses in a retrospective study evaluating 21 patients with full-thickness rotator cuff tears^21^. First, that there was no difference in clinical outcome and patient satisfaction between single tendon tears repaired through mini-open repair (MOR) or arthroscopic repair (ASR) technique and second, that stiffness would be less and recovery would be faster with ASR. However, the results of the study support the first hypothesis but not the second hypothesis^21^. In a study by Chung *et al*. evaluating postoperative stiffness in 288 patients with full-thickness rotator cuff tears, patients who underwent mini-open repair had more stiffness compared to all-arthroscopic group at the final follow-up (p = 0.02)^22^. However, there was no significant difference postoperative stiffness, pain scores, and range of motion in the two groups, in an RCT conducted by Cho *et al*.^13^.

Severud *et al*. noted that no patients in the arthroscopic group developed fibrous ankylosis, whereas 4 patients in the mini-open group developed the condition (14%), defined as failure to achieve greater than 120° forward flexion by 12 weeks postoperatively. The lower incidence of fibrous ankylosis favors the all-arthroscopic technique. A trend for better early motion was also noted in the all-arthroscopic group^19^.

Kose *et al*. reported preference of mini-open repair due to its low cost and high patient satisfaction, while also providing similar results to arthroscopic surgery^7^.

No statistically significant improvement was observed at six months in SF-36 general health, role-emotional, and mental health, in a retrospective study of 65 patients treated with arthroscopic rotator cuff repair and 63 treated with mini-open rotator cuff repair^4^. Similarly, in a case-control study design to report on 52 patients treated with either technique, the SF-36 was not significantly different postoperatively between the two groups^23^. However, in a retrospective study conducted by Osti *et al*., evaluating the two techniques in 64 patients with rotator cuff tears less than 3 cm, postoperative assessment showed a statistically significant improvement in the self-administered SF-36 scores from the preoperative values at 6 months^8^. The differences could be due to patient selection in individual studies, further, Osti *et al*. compared only rotator cuff tears with similar size and similar fixation (suture anchor)^8^.

### Limitations

The review includes both RCTs and retrospective studies, with more number of studies having retrospective study design. However, this may be due to the lack of RCT studies conducted in this area, as an unbiased methodology was used for study selection and inclusion irrespective of the study design. There were also differences in time to follow up postoperatively, with studies ranging from 6 months^4^,^5^,^13^ to 50.6 months^23^. Further, we did not investigate the impact of tear size on the outcomes, with population consisting of patients with partial-thickness rotator cuff tears less than 3 cm and full-thickness rotator cuff tears larger than 3 cm. Both single-row and double-row fixation techniques have been widely used for rotator cuff tears. Differentiation based on the use of fixation techniques was not investigated in this review, due to limitation of evidence reporting the impact of the techniques. However, in a meta-analysis it has been found that double-row fixation technique is associated with increase in post-operative rotator cuff integrity and improved clinical outcomes, especially in patients with tears larger than 3 cm^24^,^25^,^26^. Also, arthroscopic procedures were performed during the transition from mini-open to all-arthroscopic techniques; consequently, this occurred early in the learning curve in majority of the studies.

## Conclusion

In conclusion, arthroscopy repair and mini-open repair are associated with similar clinical outcomes. The choice of the operating technique depends upon the tear size and surgeon’s preference. Future research should focus on tear patterns, size, degree of delamination, mobility, and outcomes from surgical repair.

## Additional Information

**How to cite this article**: Huang, R. *et al*. Systematic Review of All-Arthroscopic Versus Mini-Open Repair of Rotator Cuff Tears: A Meta-Analysis. *Sci. Rep.* **6**, 22857; doi: 10.1038/srep22857 (2016).

## Figures and Tables

**Table 1:** Details of included studies.

Study	Year	Study type	Group	Country	Sample size	F/M	Mean age	Follow-up
Cho *et al*.	2012	RCT	ASR	South Korea	30	13/17	55.5y	6m
			MOR		30	13/17	56.2y	6m
Chung *et al*.	2013	Prospective	ASR	South Korea	225	160/128	59.5y	22.8m
			MOR		41			
			OP		22			
Colegate-Stone *et al*.	2009	Retrospective	ASR	United Kingdom	92	48/44	57y	24m
			MOR		31	15/16	62y	24m
Kang *et al*.	2007	Retrospective	ASR	USA	65	NG	NG	6m
			MOP		63			6m
Kasten *et al*.	2011	RCT	ASR	Germany	17	8/9	60.1y	6m
			MOR		17	5/12	60.1y	6m
Kim *et al*.	2003	Retrospective	ASR	South Korea	42	15/27	55y	39m(24–72)
			MOR		34	22/12	55y	39m(24–72)
Kose *et al*.	2008	Retrospective	ASR	Turkey	25	18/7	55y	31.20m
			MOR		25	21/4	62y	21.56m
Liem *et al*.	2007	Retrospective	ASR	Germany	19	3/16	61.9y	25.0m
			MOR		19	3/16	62.1y	17.6m
Nové-Josserand *et al*.	2011	Retrospective	ASR	France	154	71/183	50.5y	NG
			MOR					
Osti *et al*.	2010	Retrospective	ASR	Italy	32	17/15	56.1y	30.6m
			MOR		32	14/18	56y	31m
Pearsall *et al*.	2007	Prospective	ASR	USA	25	14/11	58y	50.6m
			MOR		27	17/10	55y	50.6m
Sauerbrey *et al*.	2005	Retrospective	ASR	USA	26	10/16	56y	19m
			MOR		28	12/16	57y	33m
Severud *et al*.	2003	Retrospective	ASR	USA	35	NG	NG	44.6m
			MOR		29			44.6m
Verma *et al*.	2006	Retrospective	ASR	USA	38	16/22	59.4y	24m
			MOR		33	10/23	60.7y	24m
Warner *et al*.	2005	Retrospective	ASR	USA	9	4/5	53y	44m
			MOR		12	4/8	55y	44m
Youm *et al*.	2005	Retrospective	ASR	USA	42	NG	60y	37.6m
			MOR		42		59y	37.6m
Zhang *et al*.	2014	RCT	ASR	China	55	27/28	53.9y	29.4m
			MOR		53	26/27	54.2y	29.4m
Zwaal *et al*.	2013	RCT	ASR	The Netherlands	47	18/29	57.2y	56w
			MOR		48	20/28	57.8y	56w

RCT: Randomized controlled trial; ASR: Arthroscopic Repair; MOR: Mini-open repair; Y: Years; M: Months; W: Weeks.

**Table 2:** Outcome measures in the meta-analysis of comparisons between all arthroscopic and mini-open cuff tear repair.

Outcome	SMD (95% CI); p-value	Heterogeneity	*I*^*2*^*%*	Number of patients	Number of studies
Abduction	1.174 (−1.019, 3.367); p = 0.294	<0.0001	94.7	104	2studies (Kasten 2011, Verma 2006)
ASES score	0.136 (−0.068, 0.340); p = 0.192	0.599	0.0	372	5studies (Kasten 2011, Kim 2003, Verma 2006, Youm 2005, Zhang 2014)
Constant score	0.865 (0.109, 1.621); p = 0.025	0.005	81.3	178	3studies (Kasten 2011, Kose 2008, Zwaal 2013)
Constant score (sensitivity analysis)	0.477 (0.039, 0.915); p = 0.033	0.366	0.0	83	2 studies (Kasten 2011, Kose 2008)
DASH	−0.013 (−0.275, 0.250); p = 0.924	0.935	0.0	223	2studies (Kang 2007, Zwaal 2013)
External rotation	0.740 (−0.426, 1.905); p = 0.213	<0.0001	95.6	323	5studies (Cho 2012, Kasten 2011, Osti 2010, Verma 2006, Zwaal 2013)
External rotation (sensitivity analysis)	0.065 (−0.268, 0.398); p = 0.703	0.192	36.7	228	4studies (Cho 2012, Kasten 2011, Osti 2010, Verma 2006)
Forward flextion	0.608 (−0.506, 1.722); p = 0.285	<0.0001	95.3	323	5 studies (Cho 2012, Kasten 2011, Osti 2010, Verma 2006, Zwaal 2013)
Forward flextion (sensitivity analysis)	0.039 (−0.221, 0.299); p = 0.770	0.660	0.0	228	4 studies (Cho 2012, Kasten 2011, Osti 2010, Verma 2006)
Internal rotation	0.058 (−0.231, 0.347); p = 0.694	0.761	0.0	185	3 studies (Kose 2008, Osti 2010, Verma 2006)
SF-36 (bodily pain)	0.044 (−0.239, 0.327); p = 0.759	0.608	0.0	192	2 studies (Kang 2007, Osti 2010)
SF-36 (role-physical)	−0.023 (−0.377, 0.331); p = 0.898	0.227	31.5	192	2 studies (Kang 2007, Osti 2010)
SST	−0.171 (−0.620, 0.278); p = 0.455	0.121	58.4	199	2 studies (Kang 2007, Verma 2006)
UCLA score	0.165 (−0.166, 0.497); p = 0.328	0.033	61.8	383	5 studies (Kim 2003, Kose 2008, Osti 2010, Youm 2005, Zhang 2014)
VAS (function)	−0.375 (−0.968, 0.217); p = 0.214	0.002	84.3	299	3 studies (Kang 2007, Kim 2003, Zwaal 2013)
VAS (function) (sensitivity analysis)	−0.084 (−0.359, 0.192); p = 0.551	0.648	0.0	204	2 studies (Kang 2007, Kim 2003)
VAS (pain)	−0.206 (−0.775, 0.364); p = 0.479	<0.0001	88.5	460	6 studies (Cho 2012, Kang 2007, Kasten 2011, Kim 2003, Verma 2006, Zwaal 2013)

ASES: American Shoulder and Elbow Surgeons’ Scoring Survey; SST: Simple Shoulder Test; UCLA: University of California, Los Angeles scoring scale.
